# Noninvasively measured immune responses reflect current parasite infections in a wild carnivore and are linked to longevity

**DOI:** 10.1002/ece3.7602

**Published:** 2021-05-07

**Authors:** Susana C. M. Ferreira, Miguel M. Veiga, Heribert Hofer, Marion L. East, Gábor Á. Czirják

**Affiliations:** ^1^ Department of Ecological Dynamics Leibniz Institute for Zoo and Wildlife Research Berlin Germany; ^2^ Department of Veterinary Medicine Freie Universität Berlin Berlin Germany; ^3^ Department of Biology, Chemistry and Pharmacy Freie Universität Berlin Berlin Germany; ^4^ Department of Wildlife Diseases Leibniz Institute for Zoo and Wildlife Research Berlin Germany; ^5^ Present address: Division of Computational Systems Biology Centre for Microbiology and Environmental Systems Science Vienna Austria

**Keywords:** age, fitness components, immune response, immunoglobulins, mucin, noninvasive, parasite infection, spotted hyena

## Abstract

Host immune defenses are important components of host–parasite interactions that affect the outcome of infection and may have fitness consequences for hosts when increased allocation of resources to immune responses undermines other essential life processes. Research on host–parasite interactions in large free‐ranging wild mammals is currently hampered by a lack of verified noninvasive assays. We successfully adapted existing assays to measure innate and adaptive immune responses produced by the gastrointestinal mucosa in spotted hyena (*Crocuta crocuta*) feces, including enzyme‐linked immunosorbent assays (ELISAs), to quantify fecal immunoglobulins (total IgA, total IgG) and total fecal O‐linked oligosaccharides (mucin). We investigated the effect of infection load by an energetically costly hookworm (*Ancylostoma*), parasite richness, host age, sex, year of sampling, and clan membership on immune responses and asked whether high investment in immune responses during early life affects longevity in individually known spotted hyenas in the Serengeti National Park, Tanzania. Fecal concentrations of IgA, IgG, and mucin increased with *Ancylostoma* egg load and were higher in juveniles than in adults. Females had higher mucin concentrations than males. Juvenile females had higher IgG concentrations than juvenile males, whereas adult females had lower IgG concentrations than adult males. High IgA concentrations during the first year of life were linked to reduced longevity after controlling for age at sampling and *Ancylostoma* egg load. Our study demonstrates that the use of noninvasive methods can increase knowledge on the complex relationship between gastrointestinal parasites and host local immune responses in wild large mammals and reveal fitness‐relevant effects of these responses.

## Graphical abstract

Host immune defenses are important components of host–parasite interactions that affect the outcome of infection and may have fitness consequences when increased allocation of resources to immune responses undermines other essential life processes. We adapted existing assays to measure innate and adaptive immune responses using fecal samples from spotted hyenas (*Crocuta crocuta*). We investigated the associations between parasite infections, immune responses, and longevity modulated by ecological and life‐history factors. Our findings increase knowledge on the complex relationship between gastrointestinal parasites and the immune responses of their host and reveal fitness‐relevant effects of immune responses to infection.

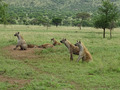

## 1 INTRODUCTION

Gastrointestinal parasites are ubiquitous in wild populations and can have negative fitness consequences for their hosts (Schmid‐Hempel, 2011). A wide range of factors, such as host age, sex, life‐history stage, parasite community composition, and infection intensity, may shape host immune phenotypes, which can change throughout host life span (Cattadori et al., 2005; Nussey et al., 2012; Watson et al., 2016). Although studies on laboratory models provide an important mechanistic understanding of the immune system and the effect of parasites on various immune responses, knowledge on this topic in free‐ranging mammals is limited (but see: Clerc et al., 2019; Graham et al., 2010; Nussey et al., 2014). Results in free‐ranging mammals may not conform to findings from laboratory studies (Abolins et al., 2018; Rosshart et al., 2019; Tian et al., 2015).

Effective innate immune barriers and adaptive immune responses to infections are crucial for the survival and reproductive success of hosts (Celi et al., 2019; Hayward et al., 2019), but these also entail energetic costs (Lochmiller & Deerenberg, 2000). Life‐history theory predicts resource allocation trade‐offs during costly life stages when food resources are limited (Sheldon & Verhulst, 1996). Investment in immune responses can divert resources from growth and reproduction (Cressler et al., 2014), whereas reduced investment in immune responses can result in increased parasite infection (Schmid‐Hempel, 2011; Sheldon & Verhulst, 1996). Immune responses are therefore probably context‐dependent and not necessarily maintained at the maximum possible level (Viney et al., 2005). As individuals vary in their investment of resources in different immune responses and the effectiveness of immune responses to parasite infection may affect survival and reproductive success (Froy et al., 2019; Graham et al., 2010; Hayward et al., 2014), knowledge of individual immune responses to infections is essential to understand complex host–parasite interactions and their impact on host fitness (Graham et al., 2011).

Hosts defend themselves from parasites by a combination of different strategies. Immune responses are complex, involving numerous effectors with various functions activated by different pathogens. For this reason, it is important to select appropriate immune markers for the host species and pathogens to be studied (Garnier & Graham, 2014) and to measure several components of the immune system (Pedersen & Babayan, 2011). When reagents and immunoassays are not available for a wild study species, these can be developed by modifying assays for related domesticated or laboratory model species or humans (Flies et al., 2012; Garnier & Graham, 2014). Such modified immunoassays require validation before adding them to a species‐specific immunoassay “toolbox” (Staley et al., 2018).

Fecal measures of immunity mostly represent immune responses by the host gastrointestinal mucosa to gastrointestinal pathogens (Watt et al., 2016). As feces can be collected repeatedly from individually known hosts without disturbing the animal, they facilitate the simultaneous assessment of parasite loads and immune responses, and in studies spanning host generations, their effects on components of fitness (Hayward et al., 2019). These advantages have prompted recent interest in the application of fecal immunological assays to wildlife (e.g., Albery et al., 2020; Dibakou et al., 2019; Gesquiere et al., 2020; Lantz et al., 2018). Several components of mucosal immunity have the potential to be measured in feces, including mucosal antibodies, mucins, and lysozyme (Table 1). Mucosal antibodies are important components of adaptive immunity that recognize and neutralize pathogens (Staley et al., 2018; Woof & Mestecky, 2005). IgA is the most abundant immunoglobulin isotype actively transported to the intestinal lumen (Russell et al., 2015), and as IgA is both stable during long‐term storage (Lantz et al., 2018; Peters et al., 2004) and heat‐tolerant (Gesquiere et al., 2020), it is a suitable immune measure for research in remote locations. Other immunoglobulins (IgM, IgG, or IgE) also occur at the intestinal mucosal barrier, but normally only small amounts of IgG diffuse into mucosal sites from plasma (Woof & Mestecky, 2005).

**TABLE 1:** Characteristics of gastrointestinal immune measures and functionality of these effectors using examples from the literature

	Mucosal immunity			
	Mucins	Total IgA	Total IgG	Total IgM	Lysozyme
Functional classification	Constitutive	Induced	Induced	Induced	Constitutive
Type of immunity	Innate	Adaptive (humoral‐mediated immunity)	Adaptive (humoral‐mediated immunity)	Adaptive (humoral‐mediated immunity)	Innate
Mechanism	Biophysical barrier; trapping of pathogens; facilitation of nematode expulsion	Recognize and neutralize pathogens; receptor blockade	Recognize and neutralize pathogens; complement and Fc‐mediated uptake	Recognize and neutralize pathogens	Activation of bacterial aggregation; blocking of bacterial adherence to the gut wall
References	McGuckin et al. (2011); Hasnain et al. (2011)	Woof and Mestecky (2005); Macpherson et al. (2008); Staley et al. (2018)	Woof and Mestecky (2005)		Hajishengallis and Russell (2015); Ragland and Criss (2017)
Ecological studies of free‐ranging mammals	–	Soay sheep: Hayward et al. (2019); Nussey et al. (2014); Watt et al. (2016); Chimpanzees: Lantz et al. (2018) House mouse: Abolins et al. (2017, 2018) Wood mice: Clerc et al. (2018); Clerc et al. (2019) Red deer: Albery et al. (2020); Baboon: Gesquiere et al. (2020) African equids: Tombak et al. (2020)	Soay sheep: Hayward et al. (2019); Watt et al. (2016)	Soay sheep: Hayward et al. (2019); Watt et al. (2016)	–

Abbreviation: FEC, fecal egg count (of parasite eggs in host feces).

We initially sought to modify and validate five immune assays for application to fecal samples from wild spotted hyenas (*Crocuta crocuta*). These included three immunoglobulins (total IgA, IgG, and IgM), mucins, and lysozyme. Validation included an evaluation of the analytical properties of the ELISAs and the fluorometric mucin assays, a comparison of systemic immune measures with fecal measures using matched blood and fecal samples from the same individuals on the day they were anesthetized with the expectation of at best a weak correlation (see Watt et al., 2016), and finally an assessment of immune measures during an 18‐day period after anesthesia in one captive animal. In this period, we expected immune measures to decline given that anesthesia elevates glucocorticoids in spotted hyenas and other mammals (Goymann et al., 1999; Sapolsky, 1982), which can decrease immune function in addition to any effect of the anesthetic per se on immune function (Kurosawa & Kato, 2008; Schneemilch et al., 2005). The process of validation revealed that fecal total IgA, IgG, and mucin were the most appropriate for our study.

The aim of our study was to apply the modified assays to: (a) investigate the association between gastrointestinal parasites, in particular the infection loads with an energetically costly hookworm (*Ancylostoma*), mucosal immune measures, and host and ecological factors of individually known wild spotted hyenas, and (b) determine the effect of immune measures on the fitness of young juvenile spotted hyenas as measured by their longevity. We reasoned that a negative relationship between parasite load and mucosal immune response suggests that the immune response is protective and provides resistance against parasite infection load. In sheep, IgA mainly from the intestine, IgG1, and IgE were negatively associated with gastrointestinal parasites (McRae et al., 2015). Similarly, fecal IgA in hamsters was negatively associated with hookworms (Bungiro et al., 2008). In this case, a positive relationship between the intensity of mucosal immune response and fitness is expected, as seen in the Soay sheep of St. Kilda, in which gastrointestinal parasite‐specific antibodies from plasma are negatively linked to gastrointestinal parasite infection loads and positively associated with winter survival (Sparks et al., 2018). By contrast, a positive relationship between a mucosal immune response and parasite infection load indicates that the intensity of the immune response increases with the severity of parasite infection. This seems to be the case seen by the positive association between infection loads of gastrointestinal parasites and fecal IgA in domestic donkeys (*Equus africanus asinus*; Tombak et al., 2020), and total fecal IgM in the Soay sheep (Hayward et al., 2019). In this case, the immune measure should have a positive relationship with parasite load and a negative relationship with survival (Graham et al., 2011).

Based on known sex differences in immune profiles of some mammals (Flies et al., 2016; Kelly et al., 2018), we expected adult females to have generally higher immune responses than adult males. Previous studies revealed differences between juveniles and adults in terms of gastrointestinal parasite loads and parasite richness in spotted hyenas (East et al., 2013; East et al., 2015; Ferreira et al., 2019) and in microbiome diversity and composition (Heitlinger et al., 2017; Rojas et al., 2020). These findings are consistent with the evidence that adaptive immunity in mammals develops as age increases (Cattadori et al., 2005; Watt et al., 2016). Hence, we expect changes in gastrointestinal infections with age to affect concentrations of mucosal immune responses.

## 2 MATERIALS AND METHODS

### 2.1 Sample collection

**FIGURE 1:**
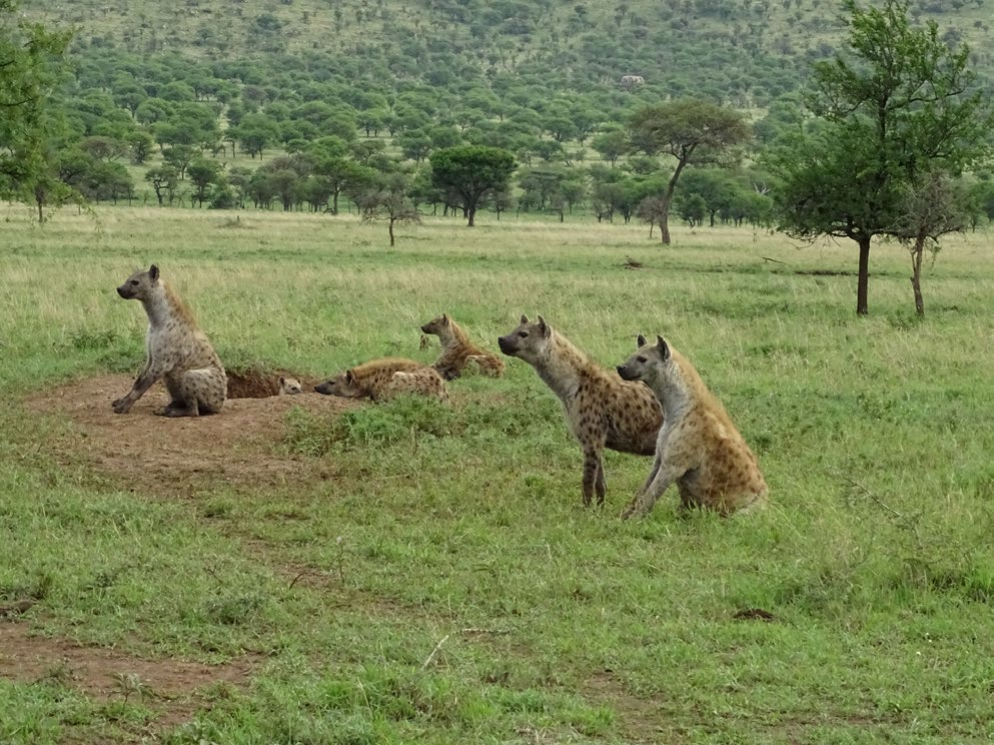
Spotted hyenas at a communal den in the Serengeti National Park, Tanzania. Photo by Susana C. M. Ferreira

We collected fresh fecal samples from individually known spotted hyenas (80 juveniles; 94 adults) that were part of long‐term research on three clans in the Serengeti National Park (NP), in northwest Tanzania throughout the years 2009 to 2017 (Ferreira et al., 2019) (see Figure 1). Fecal samples were collected immediately after defecation and stored on cool packs in the field. Samples were mechanically mixed and aliquots stored in a 4% formalin solution at room temperature for gastrointestinal fecal egg counts (East et al., 2013; see below). Aliquots for immune assays were stored frozen at −10°C until transported frozen to the Leibniz Institute for Zoo and Wildlife Research (IZW) in Berlin, Germany, where they were stored at −80°C until analysis. Not all samples could be used for all assays; thus, we report individual sample sizes used in each analysis. To compare immune measures from serum/plasma with those from feces, we used matched samples obtained on the same day from nine spotted hyenas in the Serengeti NP. Serum and plasma samples were mostly collected when animals were anesthetized for the removal of wire snares (Hofer et al., 1993). Samples for parasite fecal egg counts were not available for these nine animals. We used fecal samples from four spotted hyenas kept at the Tierpark Berlin, Germany, to optimize immune protocols. To test for an effect of anesthesia on the immune response, we used fecal and serum samples from one adult female spotted hyena at DierenPark Amersfoort, the Netherlands. Fecal samples from this animal were obtained on the day of anesthesia (day 0) and on days 6, 9, 16, and 18 thereafter. All fecal samples from captive hyenas were collected by zookeepers. Samples were stored at −20°C and transported to the IZW on dry ice, where storage was the same as for samples from wild animals.

### 2.2 Life‐history variables of free‐ranging study animals

Individuals were aged to an accuracy of 1 week (e.g., Hofer & East, 2003) and classified as juveniles if less than two years old (730 days) when sampled or adults if older. Cubs were sexed as described in Frank (1986). Longevity was scored as an exact value if the death date was known or as a right‐censored value if the individual was still alive at the end of the study (31 July 2018). Longevity was scored as the age of last sighting and as right‐censored in the case of natal males that dispersed to other clans. The longevity of individuals that died of unnatural causes (road kills and wire snares) was also scored as right‐censored.

### 2.3 Fecal egg/oocyst counts and parasite richness

We identified parasite taxa and determined fecal egg counts using the McMaster egg flotation technique, as previously described (Ferreira et al., 2019). We used 2 grams of fecal sample for this assay, resulting in the detection limit of 25 eggs per gram. Results are presented as number of eggs per gram of feces. Parasite richness is included as the count of the presence of parasite taxa (egg/oocyte identification) other than *Ancylostoma*. Each parasite taxon was included in ordination analyses in terms of abundance.

### 2.4 Immune measure quantification

For quantification of fecal immunoglobulins, we modified the sandwich ELISA of Tress et al. (2006) using a saline extract from lyophilized (freeze‐dried) fecal samples. In order to measure fecal oligosaccharides released from mucin, we adapted the protocol used by Bovee‐Oudenhoven et al. (1996) and Crowther and Wetmore (1987), which discriminates between O‐linked glycoproteins and N‐linked glycoproteins. We adapted the lysoplate assay of Osserman and Lawler (1966) previously applied to samples from wild vertebrate species (ejaculate: Rowe et al., 2013; serum: Heinrich et al., 2017) for measuring fecal lysozyme concentration using fecal saline extract. Detailed descriptions of the saline extraction and immune assays are in the Supplementary Material. Both fecal egg/oocyst counts and immune assays were done blind with respect to individual identity of hyenas.

### 2.5 Analytical validation

As commercially available IgA, IgG, or IgM from spotted hyenas or domestic cats were not available, it was not possible to quantify immunoglobulins in absolute terms. The results are thus presented as relative units (RU). Standard curves for each assay were obtained using a pool of 72 samples. To establish the working range of measurements, four random samples were diluted five times each and these were measured in eight different runs, on different days. When compared to a calibration curve, the coefficient of variation (CV) increased at high and low concentrations. Thus, the working range was defined as the interval of values with CVs below 20%.

For an analytical validation of the ELISAs, we measured the sensitivity, precision, reproducibility, and linearity of results. Sensitivity is the minimum concentration that can be reliably estimated and corresponds to three standard deviations above the mean optical density of blanks repeatedly measured (*n* = 18). The quality controls (QCs) were used to calculate the intra‐assay and interassay variation, which are a measure of the precision and reproducibility of the assay, respectively. Each sample was run within each assay 10 times, and 8 different assays were performed on different days, in duplicate. Intra‐assay and interassay CVs were deemed acceptable when <5% and <20%, respectively. The linearity of the assay was assessed by checking whether diluted measurements produced similar results after accounting for dilution (dilutional parallelism), by diluting twofold each of the two QCs four times. The criterion for evidence of linearity was set as <20% of the ratio of observed to expected values multiplied by 100.

### 2.6 Data analyses

All data analyses and statistical models were run using R version 3.6.3 (R Development Core Team, 2020). Unless otherwise stated, all statistical tests were two‐tailed.

The method to fit the standard curves for each assay was chosen based on the standard deviation of each model and visual inspection of residuals. Standard curves were fitted using package “calibFit” (Haaland et al., 2013). We fitted standard curves for the (a) ELISAs using the log parameterized four‐parameter logistic regression; (b) fluorometric (mucin) assay using the log parameterized four‐parameter logistic regression with the power of the mean method; and (c) lysoplate assay (lysozyme) using the log parameterized two‐parameter logistic regression with the power of the mean method.

We explored the relationship between parasite measures, immune measures, age class, sex, year of sampling, and clan membership in two steps. First, we analyzed the influence of *Ancylostoma* egg load on individual immune measures, since *Ancylostoma* is a costly parasite in spotted hyenas, with evidence of negative effects on components of fitness (East et al., 2015; Ferreira et al., 2019). For this purpose, we analyzed in separate models the relationship between IgA, IgG, and mucin as response variable and *Ancylostoma* egg load, parasite richness, sex, age class, the interaction between age class and sex to test whether any effect of sex is more pronounced in adults than juveniles, the interaction between *Ancylostoma* egg load and age class to test whether the effect of *Ancylostoma* egg load is more pronounced in juveniles than adults, and year of sampling and clan membership (as explanatory variables). We applied generalized linear models using the negative binomial distribution available in package “MASS” (Venables & Ripley, 2002) with a log link function (Hilbe, 2011). The significance of each predictor was assessed by using log‐likelihood ratio tests (LRT) that compared each full model with an alternative model in which the respective predictor was removed. The significance of *Ancylostoma* egg load, sex, and age class was determined with a reduced model in which the main effects and interactions were removed. The significance of each interaction was determined with a reduced model that contained the main predictors but not the respective interactions. The global goodness of fit was assessed by comparing the full model with an intercept‐only model. In preliminary models, we included an interaction between sex and *Ancylostoma* egg load as a predictor but subsequently left it out of models since it did not improve the model (ΔAIC < 2).

Second, we performed separate nonmetric multidimensional scaling (nMDS) analyses on measures of (a) parasite infection and (b) immune responses (IgA, IgG, and mucin). nMDS analyses were constructed by applying the Wisconsin double standardization, Bray–Curtis distance matrix, multiple random initial configurations, and centering and rotation using principal component analysis (Legendre & Legendre, 2012) using the package “vegan” (Oksanen et al., 2019). To access the goodness of fit, we compared the nMDS distances on the original distances and measured the *R* ^2^ of linear and nonlinear regression of both distances (Figure S1). Ellipses were calculated with function “veganCovEllipse” from the package “vegan.” Permutational MANOVA (PERMANOVA) and Bray–Curtis distance matrix (McArdle & Anderson, 2001) were used to analyze variation in parasite community by age class, sex, fecal immune measures (IgA, IgG, and mucin), year of sampling, and clan membership using the function “adonis2” (10,000 permutations) of package “vegan” (Oksanen et al., 2019). We shuffled the order of the predictors and report significant predictors that were significant regardless of their order. Parasite egg or oocyst identifications revealed the presence of eight parasite taxa (Table S1). No parasite eggs or oocytes were found in 3 out of 80 juveniles and 15 out of 94 adults; thus, they were not included in the nMDS analysis of parasite communities .

Finally, we investigated the link between each immune measure and individual longevity. For this purpose, we only included juveniles sampled before they reached 12 months of age (Ferreira et al., 2019). We applied a survival analysis and fitted Cox proportional models to look at the contribution of each immune measure as an explanatory variable controlled for age at sampling (used here as a continuous variable measured in days) and *Ancylostoma* egg load, as this parasite has a negative impact on longevity during early life on this population (Ferreira et al., 2019). Immune measures were scored as categorical variables assuming the values of high (equal to or above the median value of the immune measure) or low (below the median). Models were fitted using package “survival” (Therneau, 2020). For each model, we checked and confirmed that the assumption of proportional hazards was reasonable. In preliminary models, we scored immune measures into 3 groups: high (equal or above the minimum), moderate (between first quantile and median), and low (below the first quantile) but did not substantially improve the models (ΔAIC < 4). In the preliminary models, we also included an interaction between *Ancylostoma* egg load and immune measures, but this did not improve the models (ΔAIC < 4). Adjusted survivorship curves for the effect of IgA were calculated and plotted using package “survminer” (Kassambara et al., 2019) using the function “ggadjustedcurves” and the method “conditional” in which separate survival curves were calculated for high and low IgA concentrations after considering the effects of *Ancylostoma* egg load and age. For details of these methods, see Kassambara et al. (2019). Prediction error was calculated as the Integrated Brier Score (IBS) for each survivorship using package “pec” (Mogensen et al., 2012), with the data split method as bootstrap cross‐validations (option “BootCv,” 100 iterations allowing sampling with replacement; Figure S2).

## 3 RESULTS

### 3.1 Analytical and biological validation of IgA, IgG, and mucin assays

The summary statistics for the parallelism of the dilution curves and intra‐assay variability of ELISAs are presented in Table 2. IgM was excluded from further validation because of high intra‐assay variability. Fecal lysozyme measurements were below the limit of detection (1 µg/ml) in all (*n* = 9) but one free‐ranging adult (1.4 µg/ml) and in 3 out of 39 juveniles (1.86, 7.03, 13.84 µg/ml); thus, we refrained from further analysis of this immune measure here.

**TABLE 2:** Mean ± *SD* dilutional parallelism and intra‐assay coefficient of variation (CV) for the measurement of IgA, IgG, IgM, and mucin concentrations in feces of spotted hyenas

	Dilutional parallelism (% recovery)	Intra‐assay variability
IgA	114.60 ± 13.81	QC1: CV = 3.69, *n* = 10 QC2: CV = 3.73, *n* = 10
IgG	114.59 ± 13.69	QC1: CV = 1.78, *n* = 10 QC2: CV = 2.06, *n* = 10
IgM	n.a.	QC1: CV = 15.93, *n* = 5 QC2: CV = 24.36, *n* = 5
Mucin	115.37 ± 14.33	QC1: CV = 3.12, *n* = 10 QC2: CV = 3.05, *n* = 10

Note

IgA, IgG, and IgM are expressed in relative units (RU), and mucin is expressed in μmol oligosaccharide equivalents (OE).

Abbreviations: QC1 and QC2, two samples selected for “quality control” (see text for details); n.a., not available.

Comparison of systemic and fecal immune measures revealed a lack of correlation between total serum/plasma and fecal concentration of IgG (Spearman's *ρ* = −0.44, *n* = 9, *p* = .23) or IgA (*ρ* = −0.11, *n* = 9, *p* = .78).

**FIGURE 2:**
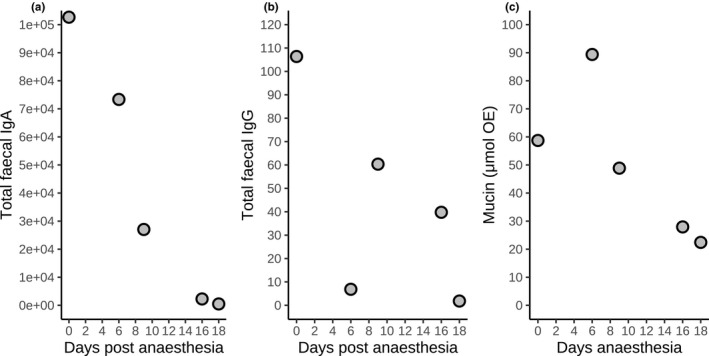
Changes in fecal immunological measures during a period of 18 days following anesthesia of a captive spotted hyena. These are total levels of fecal (a) IgG, (b) IgA, and (c) mucin. IgA and IgG are expressed in relative units (RU), and mucin is expressed in μmol oligosaccharide equivalents (OE)

As expected, fecal IgA concentration decreased (*ρ* = −1.0, *n* = 5, *p* = .02) during the 18 days following anesthesia (Figure 2a). There was a nonsignificant trend for IgG and mucin concentrations to also decline (Figure 2b–c) following anesthesia (IgG: *ρ* = −0.7, *n* = 5, *p* = .23; mucin: *ρ* = −0.9, *n* = 5, *p* = .08).

### 3.2 Factors affecting immune measures

**FIGURE 3:**
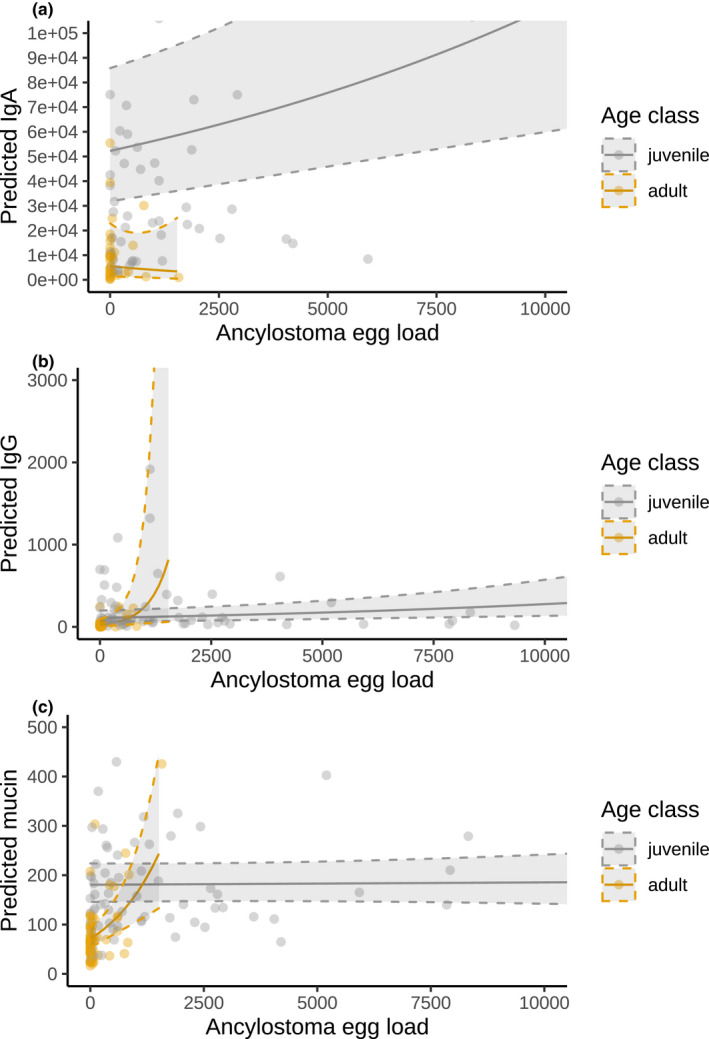
Predicted effect of age class and *Ancylostoma* egg load and age on (a) total fecal IgA (relative units [RU]), (b) total fecal IgG (relative units [RU]), and c) total fecal mucin (μmol oligosaccharide equivalents). As the effect of age class is so strong (Table 3), we reduced the window for predictions and limited these to adult immune measures for *Ancylostoma* egg load values in the raw data (0 to 1,574 eggs per gram [EPG]). Richness was the median value, the reference year was 2010, and the reference clan was Isiaka. Gray areas correspond to 95% confidence intervals, delimited by a dashed line. Points represent the raw data values. The models used for these predictions are presented in Table 3

**FIGURE 4:**
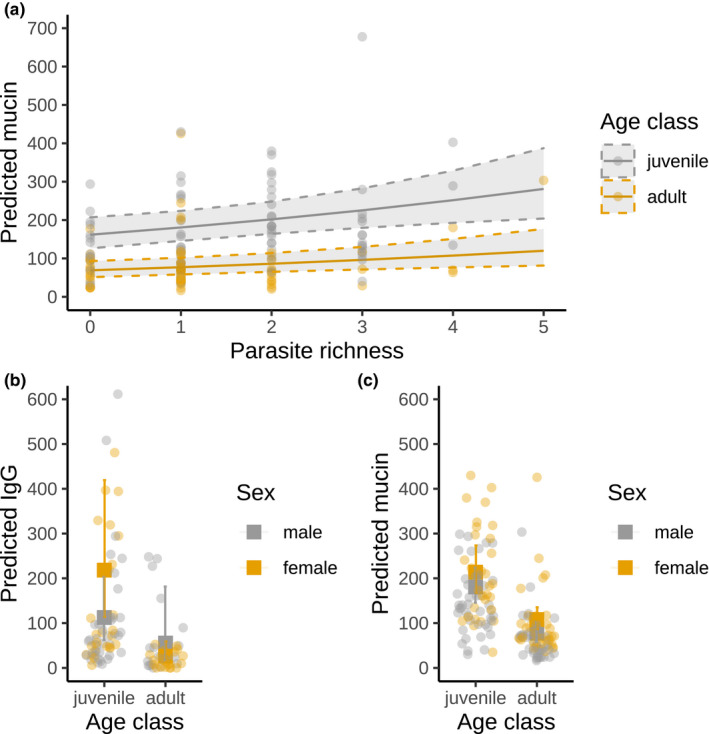
Predicted effect of (a) parasite richness and age class (juvenile/adult) on total fecal mucin (μmol oligosaccharide equivalents); (b) age class and sex (male/female) on total fecal IgG (relative units [RU]); and (c) age class and sex on the expected total fecal mucin (μmol oligosaccharide equivalents [OE]). Bars correspond to 95% confidence intervals. Reference year is 2010, and reference clan is Isiaka. The reference sex is male in (a). Parasite richness in (a) and (b) and *Ancylostoma* egg load are kept at their median values. Gray areas correspond to 95% confidence intervals, delimited by a dashed line. Points represent the raw data values. The models used for these predictions are presented in Table 3

We tested the association of IgA, IgG, and mucin measures with *Ancylostoma* egg load, parasite richness, age class, sex, year of sampling, clan membership, sex modulated by age, and *Ancylostoma* egg load modulated by age (Table 3). Fecal concentrations of IgA, IgG, and mucin all significantly increased with *Ancylostoma* egg load (Table 3, Figure 3). This effect was mediated by age class in fecal mucin (Figure 3c), with adults having both lower mucin concentrations and a steeper response to rising *Ancylostoma* load. All three immune responses to *Ancylostoma* egg loads were higher in juveniles than in adults (Table 3, Figures 3 and 4). Males had lower levels of IgG and mucin than females, and in IgG, this effect was mediated by age class (Figure 4b–c).

**TABLE 3:** Generalized linear models of factors predicting total fecal IgA, total fecal IgG, and total fecal mucin concentrations

Parameters	Estimate	*SE*	*Z*	*p*	LR stat	*p*‐value
(a) IgA: generalized linear model, G = 1,813.88, *p* < .001 (*n* = 110)						
Intercept	10.82	0.30	36.69	<.001	–	–
*Ancylostoma* egg load	0.0001	0.00002	3.09	.002	6.91	.03
Parasite richness^a^	0.04	0.10	0.41	.68	0.16	.69
Age class^b^ [adults < juveniles]	−2.25	0.72	−3.12	.002	31.88	<.001
Sex^c^ [females ≥ males]	0.43	0.26	1.64	.10	3.06	.22
Year of sampling, 2011^d^	0.43	0.26	1.66	.10	10.42	.11
Year of sampling, 2012^d^	−0.58	1.08	−0.54	.59		
Year of sampling, 2013^d^	−2.32	1.08	−2.15	.03		
Year of sampling, 2014^d^	−3.34	1.08	−3.08	.002		
Year of sampling, 2015^d^	0.46	0.61	0.76	.45		
Year of sampling, 2016^d^	0.41	0.94	0.44	.66		
Clan Mamba^e^	0.27	0.26	1.02	.31	1.73	.42
Clan Pool^e^	−0.09	0.28	−0.34	.73		
Age * *Ancylostoma* egg load	0.0004	0.0006	−0.65	.51	0.27	.60
Age * sex	−0.03	0.67	−0.05	.96	0.002	.96
(b) IgG: generalized linear model, G = 665.55, *p* < .001 (*n* = 114)						
Intercept	4.75	0.35	13.38	<.001	–	–
*Ancylostoma* egg load	0.0001	0.00003	3.24	.001	14.6	<.001
Parasite richness^a^	−0.06	0.12	−0.46	.64	0.17	.68
Age class^b^ [adults < juveniles]	−1.47	0.67	−2.19	.03	31.06	<.001
Sex^c^ [females > males]	0.66	0.31	2.13	.03	5.36	.07
Year of sampling, 2011^d^	0.01	0.30	0.04	.97	2.81	.83
Year of sampling, 2012^d^	1.48	1.29	1.15	.25		
Year of sampling, 2013^d^	0.25	0.86	0.29	.77		
Year of sampling, 2014^d^	−0.10	0.98	−0.10	.92		
Year of sampling, 2015^d^	0.40	0.59	0.68	.50		
Year of sampling, 2016^d^	−0.25	1.00	−0.25	.80		
Clan Mamba^e^	0.40	0.30	1.33	.18	3.86	.15
Clan Pool^e^	0.68	0.33	2.05	.04		
Age * *Ancylostoma* egg load	0.002	0.0001	2.24	.02	3.45	.06
Age * sex [Figure 2c]	−1.41	0.64	−2.20	.03	4.16	.04
(c) Mucin: generalized linear model, G = 751.14, *p* < .001 (*n* = 151)						
Intercept	5.09	0.13	40.34	<.001	–	–
*Ancylostoma* egg load	0.000003	0.00001	0.25	.81	17.29	<.001
Parasite richness^a^	0.11	0.04	2.84	.005	8.45	.004
Age class^b^ [adults < juveniles]	−0.93	0.15	−6.10	<.001	41.72	<.001
Sex^c^ [females > males]	0.17	0.12	1.43	.15	7.67	.02
Year of sampling, 2009^d^	−0.02	0.37	−0.06	.95	22.48	.004
Year of sampling, 2011^d^	−0.33	0.10	−3.14	.002		
Year of sampling, 2012^d^	0.16	0.49	0.33	.74		
Year of sampling, 2013^d^	−0.16	0.26	−0.62	.54		
Year of sampling, 2014^d^	−0.67	0.24	−2.85	.004		
Year of sampling, 2015^d^	−0.48	0.14	−3.35	.001		
Year of sampling, 2016^d^	−0.49	0.21	−2.31	.02		
Year of sampling, 2017^d^	−0.14	0.28	−0.51	.61		
Clan Mamba^e^	0.01	0.10	0.14	.89	0.05	.97
Clan Pool^e^	0.02	0.11	0.23	.82		
Age * *Ancylostoma* egg load [Figure 2b]	0.001	0.0002	3.86	.001	17.42	<.001
Age * sex	0.17	0.18	0.96	.34	0.89	.34

Note

Explanatory variables include the *Ancylostoma* egg load, parasite richness (the number of gastrointestinal taxa per individual apart from *Ancylostoma*), age class (juvenile or adult), sex (male or female), year of sampling (2009/2011–2017), clan membership (Isiaka, Mamba, Pool), and the interactions between sex and age class and *Ancylostoma* egg load and age class. Each model assumed a negative binomial distribution. Shown are the parameter estimates from the full model, with the corresponding standard error (SE), Z‐statistics, and corresponding *p*‐value (*p*); and the log‐likelihood ratio test (LRT) values (LR stat) with the associated *p*‐values (*p*‐value) of the difference between the full model and the reduced model. In particular, we report the LRT of the main for *Ancylostoma* egg load, age class, and sex by comparing the full model with a reduced model in which the main effects and interactions are removed. The LRT of the corresponding interactions are calculated by comparing the full model with a reduced model in which we keep the main effects but remove the interaction.

^a^ Presence of one or more of the following taxa: *Diphyllobothrium*, *Cystoisospora*, *Dipylidium*, Taeniidae, *Trichuris*, Spirurida, Mesocestoides.

^b^ Reference age class is adult.

^c^ Reference sex is female.

^d^ Reference year is 2010.

^e^ Reference clan is Isiaka.

### 3.3 Factors affecting parasite community

**FIGURE 5:**
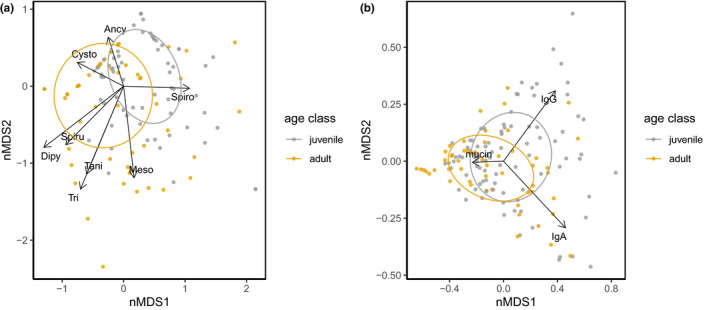
Nonmetric multidimensional scaling of parasite communities and immune measures, respectively. Each data point represents one individual, and different colors represent age classes. Circles represent covariance ellipses by age class, and arrows correspond to the variables (parasite taxa or immune measures) driving the distribution pattern. (a) Parasite communities composed of the parasite load of eight gastrointestinal taxa in performing flotations of fecal samples. Included taxa are as follows: *Ancylostoma*, *Diphyllobothrium*, *Cystoisospora*, *Dipylidium*, Mesocestoides, *Trichuris*, Taeniidae, and Spirurida. Stress value: 0.18. (b) Immune measures, composed of fecal IgA, fecal IgG, and fecal mucin. Stress value: 0.03

Ordination techniques are used to reduce dimensionality in ecological, parasitological, or immunological datasets with several correlated variables by creating a small number of new uncorrelated variables termed axis (Abolins et al., 2018; Watson et al., 2016). We performed separate nMDS analyses to visualize how individuals varied in these reduced dimensions. The nMDS analysis for the parasite community (8 parasite taxa; Figure 5, Figure S1) used data from 156 individuals (79 adults and 77 juveniles; Figure 5a), and the nMDS analyses on immune profiles (fecal measures of IgA, IgG, and mucin) used data from 145 individuals with all three immune measures (54 adults and 91 juveniles; Figure 5b). Additionally, we tested the association of sex, age class, each immune measure, year of sampling, and clan membership with the parasite community using data from 69 juveniles and 24 adults (*n* = 93). Fecal IgA (PERMANOVA, *r* ^2^ = .03, *p* = .003) and age class (PERMANOVA, *r* ^2^ = .09, *p* < .001) explained a small but significant variation, but not sex (PERMANOVA, *r* ^2^ = .01, *p* = .37), fecal IgG (*r* ^2^ = .006, *p* = .83), fecal mucin (PERMANOVA, *r* ^2^ = .01, *p* = .33), year of sampling (*r* ^2^ = .05, *p* = .53), or clan membership (*r* ^2^ = .01, *p* = .78).

### 3.4 IgA concentrations during early life and longevity

**FIGURE 6:**
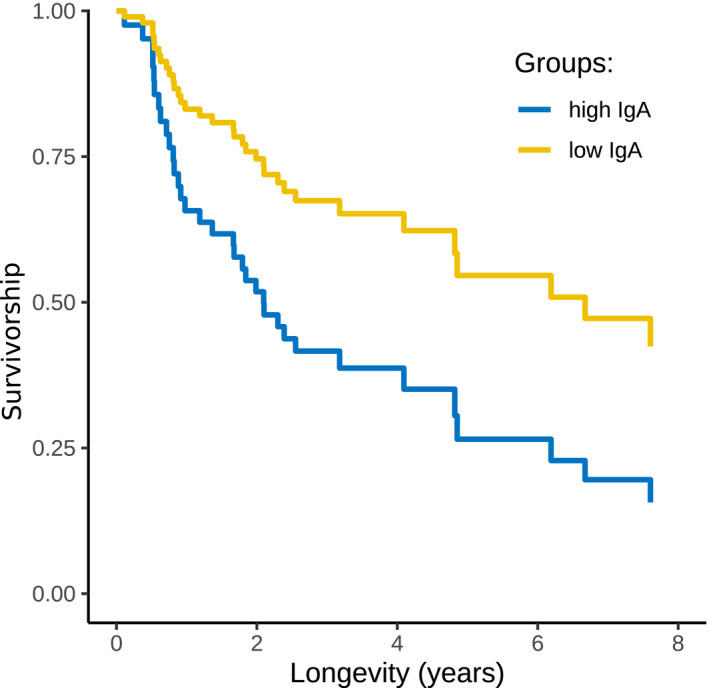
Survivorship of juvenile spotted hyenas sampled during the first 12 months of life as a function of their total fecal IgA immune response on the sampling data. Longevity indicates the length of life in years. The curves are the survivorship curves from a Cox proportional hazards model (method = “conditional”) adjusted for age and *Ancylostoma* egg load. Yellow: At time of sampling, animals had a total fecal IgA response below the median (*n* = 45). Blue: At time of sampling, animals had a total fecal IgA response above or equal to the median (*n* = 20)

We constructed 3 Cox proportional hazards models to investigate the effects of immune measures on longevity while controlling for age and *Ancylostoma* egg load. Longevity was shorter for individuals with higher fecal IgA concentrations as juveniles (high > low: *Z* = −2.45, *p* = .01) after the effects of *Ancylostoma* egg load (*Z* = 1.96, *p* = .05) and age at sampling (*Z* = −1.38, *p* = .17) were taken into consideration (survival analysis, Cox proportional hazards model, G = 14.6, *df* = 3, *p* = .002, *n* = 65, number of events = 34, juveniles younger than 12 months; Figure 6). There was no such effect of fecal IgG on longevity (*Z* = −0.68, *p* = .50) after the effect of *Ancylostoma* egg load (*Ancylostoma*: *Z* = 2.06, *p* = .04) and age (*Z* = −1.84, *p* = .07) was considered (G = 9.25, *df* = 3, *p* = .03, *n* = 65, number of events = 34). Similarly, there was no effect of mucin on longevity (*Z* = −1.34, *p* = .18) after the effect of *Ancylostoma* egg load (*Z* = 1.72, *p* = .09) and age (*Z* = −1.73 *p* = .08) was considered (G = 0.57, *df* = 3, *p* = .01, *n* = 66, number of events = 34).

## 4 DISCUSSION

In order to improve knowledge on parasite–host interactions for gastrointestinal parasite infections and gastrointestinal mucosal immune responses of hosts, we modified and validated assays for both adaptive (IgA, IgG) and innate (mucin) immune processes to epithelial cell samples collected from feces from spotted hyenas. Analyses using individual‐based measures of these components of mucosal immunity and parasite infection (parasite community, *Ancylostoma* infection load, and parasite richness) in wild spotted hyenas revealed complex interactions between these measures, host age and sex (Table 3, Figures 3, 4, and 5). Longevity of juveniles sampled during the first year of life was reduced in individuals with elevated concentrations of IgA (Figure 6). IgA concentrations were reduced during the 18 days following anesthesia (Figure 2) in one captive animal. As expected, there was no correlation between systemic and fecal levels of immunoglobulins (Watt et al., 2016), although we cannot exclude that this may be a consequence of our limited sample size.

Immune measures can be a response to or a consequence of infection with parasites (Graham et al., 2011). Studies in wild populations found complex interactions between immune measures and infection loads, lending support to particular immune measures providing resistance to parasites or reflecting parasite exposure. For instance, in wild wood mice, total fecal IgA was negatively associated with *Heligmosomoides polygyrus* and *Eimeria* infection load, suggesting a protective effect, whereas parasite‐specific IgG1 concentration was positively associated with *H. polygyrus* infection load and negatively associated with pinworm infection load (Clerc et al., 2018), suggesting that IgG1 reflects parasite exposure to *H. polygyrus* and has protective effects through cross‐reactivity to pinworms. Measuring fitness proxies, the infection load of relevant parasites and immune measures can provide rich insights into these relationships and their directionality.

*Ancylostoma* “hookworm” parasites are energetically costly to their host owing to the damage they cause to the intestinal lining and the blood they consume (Coop & Kyriazakis, 1999). Our results support a previous study demonstrating that high *Ancylostoma* infection loads in juvenile spotted hyenas reduce longevity (Ferreira et al., 2019). Similarly, in other wild mammals, high hookworm infections reduced body condition and growth rates in juveniles (Chilvers et al., 2009; Seguel & Gottdenker, 2017). These findings suggest that spotted hyenas should seek to limit the potentially detrimental effects of high *Ancylostoma* infection loads by mounting immune responses. In line with this expectation, fecal measures of IgA, IgG, and mucin concentrations were all positively associated with *Ancylostoma* egg load (Table 3) and the variation in the parasite community (8 parasite taxa) was partially explained by IgA concentration, suggesting that spotted hyenas increase resource allocation to these immune responses as a result of rising *Ancylostoma* infections. Whereas measuring total (hence specific plus unspecific) immune measures is likely to reflect parasite exposure, it is nevertheless possible that specific parasite‐induced immune measures such as parasite‐specific antibodies are designed and produced to have a protective effect.

We found evidence that juvenile spotted hyenas with high levels of fecal IgA during their first year of life had a lower longevity than juveniles with low levels of IgA (Figure 6), after *Ancylostoma* egg load and age at sampling had been accounted for. This suggests that a lower production of fecal IgA by juveniles during early life permits more body resources to be allocated to growth than would otherwise be possible. In our study population, juvenile survival increases with juvenile growth rate (Hofer & East, 2003). Our finding is in line with evidence that some immune responses in vertebrates are energetically costly and can have negative fitness consequences (e.g., Colditz, 2008; Lochmiller & Deerenberg, 2000; Mills et al., 2010).

Fecal concentrations of IgA, IgG, and mucin were higher in juvenile spotted hyenas than in adults (Table 3). In mammals, intestinal immunity develops as juvenile age increases and is qualitatively and quantitatively different from the adult immune system (Dowling and Levy, 2014; Watson et al., 2016). Increased exposure to microbes and pathogens with age also shapes the immune system (Laforest‐Lapointe & Arrieta, 2017; Simon et al., 2015). Our results revealed that juveniles had (a) a different composition of parasite communities than adults (Figure 5) and higher *Ancylostoma* infection loads than adults (Table S1), (b) higher concentrations of mucin, total fecal IgA, and IgG than adults (Table 3, Figure 4), and (c) a shallower increase in mucin concentrations with *Ancylostoma* infection loads than adults (Figure 4c). These results suggest that the immune responses of juveniles differed to those of adults probably because their immune response was immature and thus unlikely to be protective. Juvenile spotted hyenas are stationary at the clan communal den until approximately 12 months old (Hofer et al., 2016; Hofer & East, 1993). Juveniles have a lower microbiome richness and diversity than adults in our study population (Heitlinger et al., 2017) and a higher prevalence of some intestinal pathogens (East et al., 2013; Goller et al., 2013). Juveniles had high parasite prevalence and infection load of directly transmitted parasites such as *Ancylostoma* and *Cystoisospora* and low prevalence and infection load of parasites with a complex life stage that requires exposure to intermediate hosts such as Taeniidae and Spirurida. In addition, juveniles may also have higher levels of immune responses than adults because they had higher parasite loads than adults (Ferreira et al., 2019), lending further support that the measured immune responses reflect parasite infection load and contribute to differences in immune responses between age classes.

Other factors likely to influence immune responses are the behavioral, physiological, and genetic effects of sex (Metcalf et al., 2020). In mammals, adult males often have higher parasite loads than females (Poulin, 1996). Adult males more often invest in energetically costly competition to increase mating success (Zuk et al., 1990) and reduce their investment in immune responses (Rolff, 2002; but see: Stoehr & Kokko, 2006; Kelly et al., 2018). A previous study reported no effect of sex on *Ancylostoma* and *Cystoisospora* infection loads in juvenile spotted hyenas (Ferreira et al., 2019). A study of systemic immunity in spotted hyenas revealed that males had lower immune measures of serum IgG and IgM than females and increased complement‐mediated bacterial killing capacity (Flies et al., 2016). We did not find an effect of sex on fecal IgA concentrations, whereas females had higher mucin and a tendency for higher IgG concentrations than males. Interestingly, the sex differences in fecal IgG in adults were not as stark as in juveniles (Figure 4b) and adult males on average surpassed adult females in terms of IgG levels.

Stressors (Martin, 2009; Sapolsky et al., 2000) such as unpredictable or uncontrollable aversive conditions may induce physiological and behavioral responses, which may adversely affect immune responses and reduce fitness (Hofer & East, 2012). The release of stress response mediators such as glucocorticoids and neurotransmitters modulates several components of the immune system in a dose‐dependent and context‐dependent manner (Sapolsky et al., 2000), compromising immune function by the effect of anesthetic drugs on components of the immune system and by activating the hypothalamic–pituitary–adrenal axis and the autonomic nervous system (Kurosawa & Kato, 2008). Anesthesia significantly elevates glucocorticoid concentrations in captive spotted hyenas (Benhaiem et al., 2012; Goymann et al., 1999) and should therefore impair immune function. Indeed, IgA concentrations were reduced for 18 days following anesthesia in one captive animal, but there was no significant effect on IgG or mucin concentrations, although they showed a tendency to decrease (Figure 2). Studies on other species revealed that elevated concentrations of glucocorticoids are associated with reduced mucosal antibody concentrations (Alverdy & Aoys, 1991; Campos‐Rodríguez et al., 2013; Wira et al., 1990) and the depletion of goblet cells that produce mucins (Castagliuolo et al., 1996).

Our findings illustrate that noninvasive methods that quantify immune responses in wild large mammals can increase knowledge on the complex relationship between gastrointestinal parasites and the immune responses of their host and reveal fitness‐relevant effects of immune responses to infection.

## CONFLICT OF INTEREST

None declared.

## AUTHOR CONTRIBUTIONS

**Susana Carolina Martins Ferreira:** Conceptualization (equal); Data curation (supporting); Formal analysis (lead); Funding acquisition (equal); Investigation (equal); Methodology (equal); Writing‐original draft (lead); Writing‐review & editing (equal). **Miguel M. Veiga:** Funding acquisition (supporting); Methodology (supporting); Writing‐review & editing (supporting). **Heribert Hofer:** Conceptualization (supporting); Data curation (supporting); Formal analysis (supporting); Funding acquisition (equal); Investigation (supporting); Project administration (supporting); Supervision (supporting); Writing‐review & editing (supporting). **Marion L. East:** Conceptualization (equal); Data curation (lead); Funding acquisition (supporting); Investigation (equal); Methodology (supporting); Project administration (equal); Resources (equal); Supervision (equal); Writing‐original draft (equal); Writing‐review & editing (equal). **Gábor Árpád Czirják:** Conceptualization (equal); Investigation (supporting); Methodology (equal); Supervision (equal); Validation (lead); Writing‐original draft (supporting); Writing‐review & editing (supporting).

## Supporting information

Appendix S1

## Data Availability

The dataset analyzed for this study is available from the Dryad Digital Repository, https://doi.org/10.5061/dryad.sqv9s4n3s.
