# Supplementary material for: Noninvasively measured immune responses reflect current parasite infections in a wild carnivore and are linked to longevity
Source: Ecol Evol. 2021 May 7;11(12):7685–99. doi: 10.1002/ece3.7602 (PMC8216923; doi:10.1002/ece3.7602)
Supplement: Supplementary file 1 — Appendix S1 [file ECE3-11-7685-s001.docx]

**Supplementary Material**

**Table A1.** Gastrointestinal parasite taxa identified from eggs/oocysts from faecal samples of spotted hyenas. Indicated are the percentage of infected host individuals (prevalence, %), mean infection loads of infected hosts (mean intensity, number of eggs or oocysts/g faeces), mean infection load across all hosts, including the non-infected hosts with a load of 0 (mean abundance, number of eggs or oocysts/g faeces) and the ratio of the variance to the mean of abundance. We present results for juveniles (j, *n*=80), adults (a, *n*=94) and total (t, *n*=174).

| Parasite | Phylum | Prevalence | Mean intensity | Mean abundance | Ratio variance / mean abundance |
| --- | --- | --- | --- | --- | --- |
| *Ancylostoma* | Nematoda | j: 94%  a: 52%  t: 71% | j: 336614  a: 24311  t: 213204 | j: 315576  a: 12673  t: 151939 | j: 99.0  a: 5.6  t: 109.2 |
| *Diphyllobothrium* | Platyhelminthes | j: 50%  a: 67%  t: 59% | j: 1642094  a: 650599  t: 1035646 | j: 821047  a: 436040  t: 613055 | j: 395.4  a: 1641.2  t: 875.4 |
| *Cystoisospora* | Apicomplexa | j: 53%  a: 21%  t: 36% | j: 266667  a: 35125  t: 191976 | j: 140000  a: 7473  t: 68405 | j: 239.9  a: 12.9  t: 231.4 |
| *Dipylidium* | Platyhelminthes | j: 24%  a: 0%  t: 11% | j: 4078  a: 0  t: 4079 | j: 969  a: 0  t: 445 | j: 0.4  a: -  t: 0.5 |
| Taeniidae | Platyhelminthes | j: 6%  a:10%  t: 8% | j: 3000  a: 7778  t: 6071 | j: 188  a: 745  t: 489 | j: 0.3  a: 1.4  t: 1.2 |
| *Trichuris* | Nematoda | j: 8%  a: 3%  t: 5% | j: 4583  a: 3333  t: 4167 | j: 344  a: 106  t: 216 | j: 0.6  a: 0.4  t: 0.5 |
| Spirurida | Nematoda | j: 6%  a: 9%  t: 7% | j: 8000  a: 26406  t: 19326 | j: 500  a: 2247  t: 1443 | j: 1.1  a: 6.0  t: 5.2 |
| Mesocestoides | Platyhelminthes | j: 15%  a: 14%  t: 14% | j: 30625  a: 21730  t: 26000 | j: 4594  a: 3005  t: 3736 | j: 12.5  a: 5.4  t: 9.4 |

**Figure A1.** Shepard and goodness of fit diagrams of the nMDS analyses of parasite community profile and immune profile in spotted hyenas.


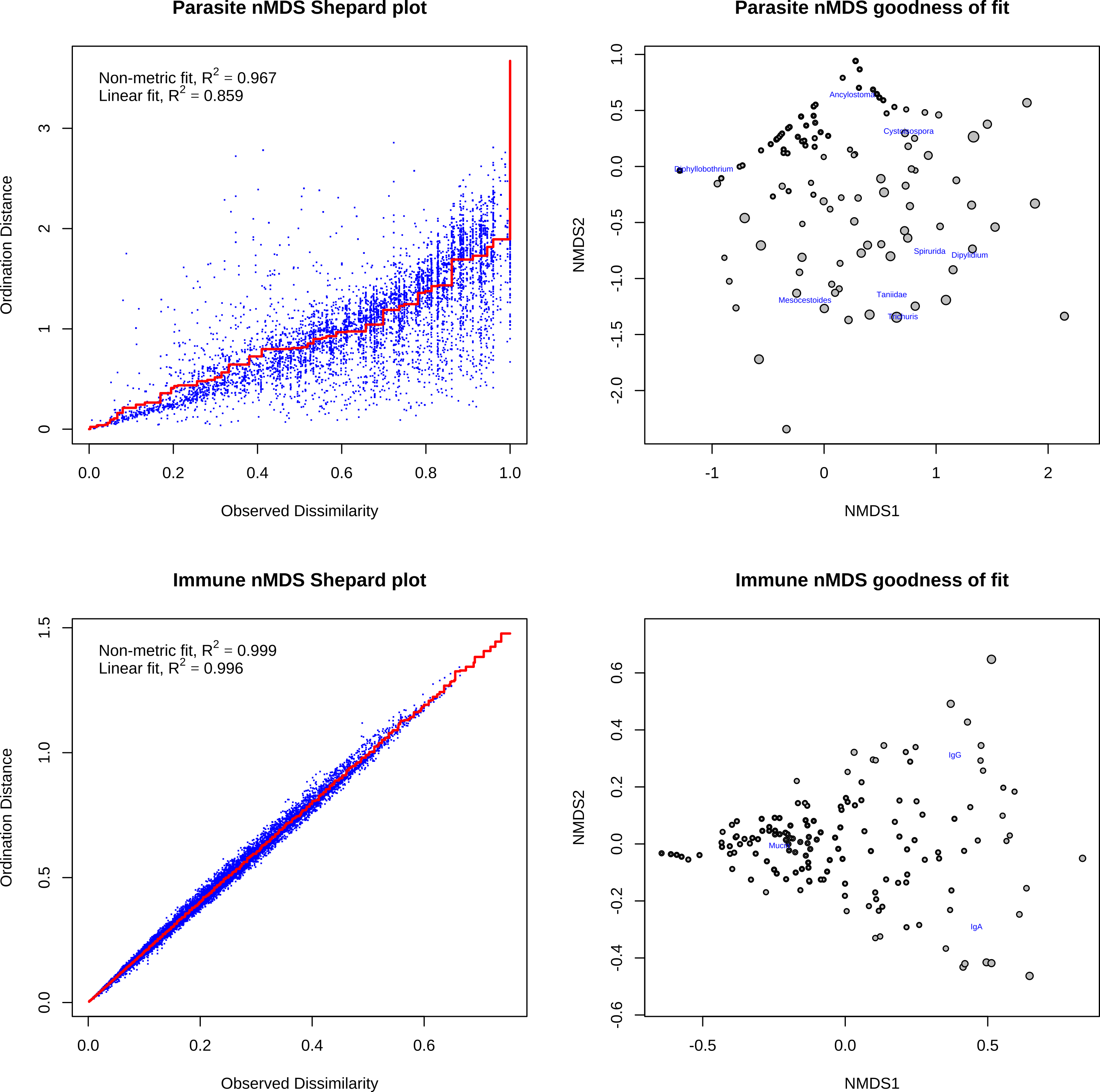


**Figure A2.** Cumulative prediction error (integrated Brier score [IBS]) from 0 to 7.6 years (2851 days) was calculated with bootstrap cross-validation across models. IBS scores were separately calculated for the models for each immune measure and their corresponding nested models. Reference model (black) had no covariate for each immune measure, limited model (green) included age and *Ancylostoma* as covariates in the model and excluded specific immune measures, full model shown in red. (a) IgA integrated IBS score from 0 to 7.6 years for reference model (black) 0.225; limited model (green) 0.225, full model (red) 0.222. (b) IgG integrated IBS score from 0 to 7.6 years for reference model (black) 0.225, limited model (green) 0.227, full model (red) 0.236. c) Mucin integrated IBS score from 0 to 7.6 years for reference model (black) 0.229, limited model (green) 0.230, full model (red) 0.234. All IBS were calculated with 100 bootstrap samples using the R package pec.

**
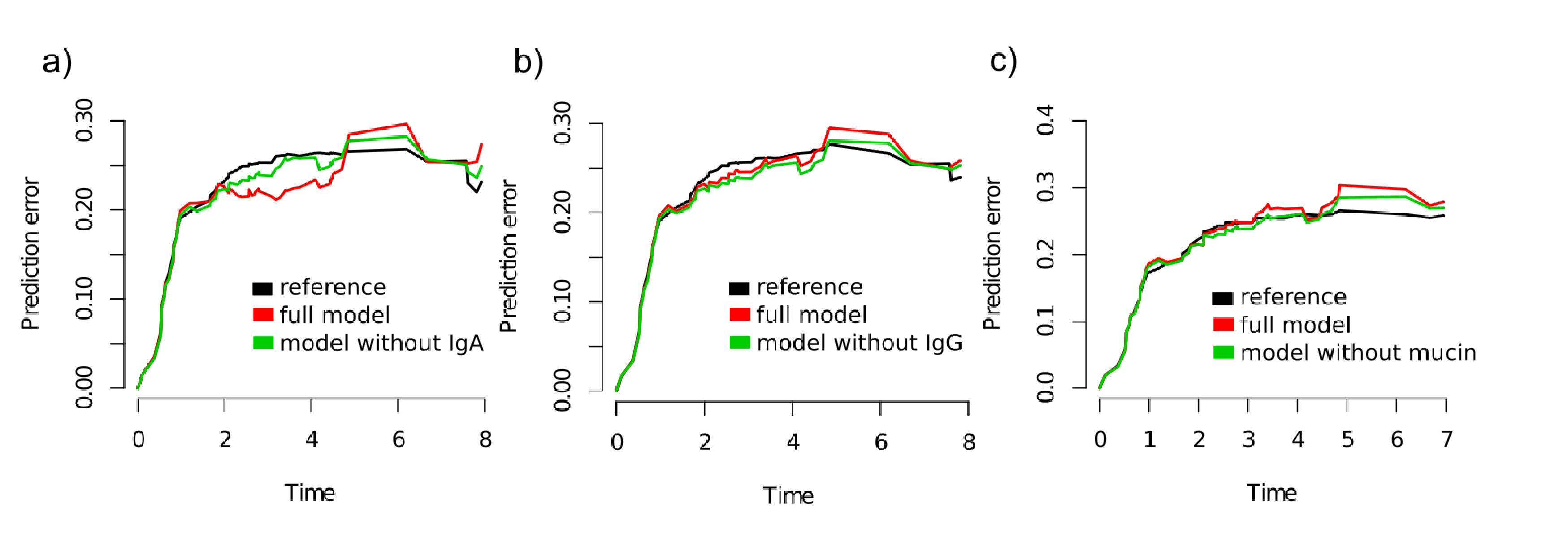
**

**Immune assay descriptions**

**Faecal saline immunoglobulin extraction**

Samples were lyophilised for 22h using a freeze drier (Epsilon1-4 LSCplus, Martin Christ GmbH, Osterode, Germany), and homogenised to a powder using mortar and pestle. Saline extraction was modified from Ferguson et al. (1995) and Peters et al. (2004). Extraction buffer (0.01M phosphate-buffered saline [PBS] (pH 7.4), 0.5% Tween-20 [Sigma-Aldrich Chemie GmbH, Taufkirchen, Germany], and 0.05% sodium azide) was added to freeze dried samples at a concentration of 10mL/g and homogenised by a combination of manual shaking and mechanical homogenisation using a vortex followed by centrifugation at 1,500G for 20 min at 5°C. The supernatant was separated and protease-inhibitor MixM (Serva Electrophoresis GmbH, Heidelberg, Germany) added at a concentration of 10µL per gram of faecal powder. The solution was mechanically homogenised with a vortex mixer and centrifuged at 10,000G for 10 min at 5°C. The supernatant was then separated and stored at -20°C until further measurements.

**ELISA for quantification of faecal immunoglobulins**

For quantification of faecal immunoglobulins we modified the sandwich ELISA of Tress et al. (2006). A grid experiment was used to determine the optimal concentrations of coating and detection antibodies and sample extracts for each assay. As purified immunoglobulins from spotted hyenas are not commercially available, the standard curves were performed using pooled faecal extracts from wild spotted hyenas. Two samples with different concentrations of IgA and IgG were selected as “quality controls” (QC) and were run with each assay. Buffer A (see below) was used as negative control and ran in each assay. The ELISA was performed as follows: A solution of 100µL of the capture antibody with a ratio of 1:200 of anti-cat IgA (Lot.A10, Novusbio, Abingdon, UK) and dilution buffer (0.05M sodium-carbonate monohydrate buffer [Na_2_CO_3_·H_2_O], pH 9.6) or 1:400 of anti-cat IgG (5210-0198, medac GmbH, Wedel, Germany) or 1:200 of anti-cat IgM (01-20-03, KPL, Maryland, USA) was added to a 96-well plate (Sarstedt, Nümbrecht, Germany) and incubated for 60 min at 37°C. Each well was then washed four times with 300µL of washing buffer (Tris-Buffered Saline, 0.05% Tween-20 [TBS-T20], pH 7.4). Nonspecific binding sites were blocked by adding 200 µL per well of blocking (Tris-Buffered Saline, 0.05% Tween-20, 10% gelatin). After 1h incubation at 37°C, each well was washed as previously described and 100µL of sample, quality controls, negative controls and standard solution added to the respective wells in a Tris-buffered saline solution (pH 7.4) containing 1% bovine serum albumin (Serva, Heidelberg, Germany) and 0.05% Tween-20 (buffer A). All samples were performed in duplicate. Results were accepted if the coefficient of variation (CV) was below 5% and within the working range previously established (see analytical validation section below).

To determine the standard curve of the IgA assay, two-fold dilutions were performed ten times, starting from a dilution of 1:320; for the IgG standard curve, two-fold dilutions were performed nine times, starting from a dilution of 1:2; for the IgM standard curve two-fold dilutions were performed nine times, starting from a dilution of 1:100. After incubation for 1h at 37°C the wells were washed as described above and 100 µL/well of detection antibody solution was added using a ratio of 1:200 conjugated anti-cat IgA (Lot.P18, Novusbio, Abingdon, UK) and buffer A, or 1:50 conjugated anti-cat IgG (5220-0369, medac GmbH, Wedel, Germany), or 1:100 conjugated anti-cat IgM (04-20-03, KPL, Maryland, USA). After incubation of 1h at 37°C, 100µL of the substrate solution was added. Substract solution was prepared by dissolving 0.01g of 3,3′, 5,5′-tetramethylbenzidine [TMB] with 1mL of dimethylsulfoxide [DMSO] and 100µL of this solution added to 10mL of 0.05M phosphate-citrate buffer (pH 5) and 3µL of 30% hydrogen peroxide [H_2_O_2_] immediately prior to use. The plate was then incubated at room temperature for 20 min and the reaction was stopped using 100µL per well of the stop solution (1M H_2_SO_4_). Plates were read at a wavelength of 450nm with the BioTek Quant Microplate reader (BioTek, Vermont, USA).

**Fluorometric (mucin) assay**

In order to measure faecal oligosaccharides released from mucin, we adapted the protocol used by Bovee-Oudenhoven et al. (1996) and Crowther & Wetmore (1987), which discriminates between O-linked glycoproteins and N-linked glycoproteins. Fresh alkaline 2-cyanoacetamide (CNA) reagent was prepared for each assay by mixing 200µL of 0.6M 2-cyanoacetamide (C_3_H_4_N_2_O) with 1mL of 0.15N NaOH. An amount of 0.5g freeze dried faeces was suspended in 500µL of PBS (1X, pH 7.4), mixed and incubated at 95°C for 10 min with constant shaking to denature glycosidases. Thereafter mucins were made soluble by incubating for 90 min at 37°C and centrifuging for 1 min at 15,000G. Of the resulting supernatant 20µL were mixed with 24µL of alkaline CNA reagent in a disposable polypropylene microtube and incubated for 30 min at 100°C. After incubation, 200µL of 0.6M borate buffer (pH 8.0) was added and the solution allowed to cool at room temperature. Of the resulting solution 100µL were transferred to a 96-well black plate for measuring fluorescence at 383nm using a fluorescence spectrophotometer (Infinite M200, TECAN, Männedorf, Switzerland) with an excitation wavelength of 336nm. Results are expressed as µmol oligosaccharide equivalents (OE). In each assay, seven two-fold dilutions starting from 1:1 of a concentration of 500µg/mL of N-acetylgalactosamine were used as standard and porcine stomach mucin (Sigma-Aldrich, Darmstadt, Germany) and two faecal sub-samples from two spotted hyenas were used as positive control and quality controls, respectively.

**Lysoplate assay**

We adapted the lysoplate assay of Osserman & Lawler (1966) previously applied to samples from wild vertebrate species (ejaculate: Rowe et al. 2013; serum: Heinrich et al. 2017). A solution with PBS (1X, pH 6.3), 1% noble agar (Sigma Aldrich; St. Louis, USA) and 25mg/100mL of lysozyme-sensitive bacteria *Micrococcus lysodeikticus* (Sigma Aldrich; St. Louis, USA, catalogue number M3770) at 56°C was poured into a sterile plate (Sarstedt, Nümbrecht, Germany) on a level surface and allowed to cool, then stored at 4°C until use. Standards were prepared using lysozyme from chicken egg white (Lot. SLBL7146, Sigma Aldrich; St. Louis, USA,), with the concentrations of 0.5, 0.8, 1, 2, 4, 8, 10 and 20µg/mL using PBS (pH 6.3). The standards and samples were added to previously punctured holes in the plate with a diameter of 5mm and incubated at 22°C for 18h. After incubation the plates were photographed and the diameter of the clear zone around each hole was measured twice. The standard curve was used with the mean of the two diameter measurements.
